# Management of Symphysis and Parasymphysis Mandibular Fractures in Children Treated with MacLennan Splint: Stability and Early Results

**DOI:** 10.5005/jp-journals-10005-1298

**Published:** 2015-08-11

**Authors:** Abhishek Khairwa, Manohar Bhat, Anupama Sharma, Rajesh Sharma

**Affiliations:** Senior Lecturer, Department of Pedodontics, Jaipur Dental College, Jaipur Rajasthan, India; Principal and Head, Department of Pedodontics, Jaipur Dental College, Jaipur Rajasthan, India; Assistant Professor, Department of Community Dentistry, Government Dental College, Jaipur, Rajasthan, India; Professor, Department of Pedodontics, Jaipur Dental College, Jaipur Rajasthan, India

**Keywords:** MacLennan splint, Mandibular fractures, Sports injury.

## Abstract

**Objective:** The aim of this study was to assess the safety and efficiency of MacLennan splint in symphysis and parasymphysis mandibular fractures in children.

**Study design:** Six patients (four boys and two girls, mean age 3 years, range between 2 and 5 years) were operated on parasymphysis fractures of children. The mean follow-up time was 12 months. MacLennan splint was applied in these case upto 3 weeks.

**Results:** Primary healing of the fractured mandible was observed in all patients. Postoperative complications were minor and transient. The outcome of the operation was not endangered. Adverse tissue reaction like infection, malocclusion, swelling and growth restrictions did not occur during observation period.

**Conclusion:** MacLennan splint is having various advantages like faster mobilization and the avoidance of secondary removal operations. Based on this preliminary results MacLennan splints are safe and efficient in the treatment of pediatric mandible fracture.

**How to cite this article:** Khairwa A, Bhat M, Sharma A, Sharma R. Management of Symphysis and Parasymphysis Mandibular Fractures in Children Treated with MacLennan Splint: Stability and Early Results. Int J Clin Pediatr Dent 2015;8(2):127-132.

## INTRODUCTION

Trauma is described as injury resulting from an external force. It is the leading problem that children are facing today.^1^ Mandibular fracture was first noticed in Edwin Smith Papyrus in 1650 BC. Pediatric bone fracture is a particular pathology, because it occurs on a rapidly growing bone. In the child’s face, the most common site of fracture is the nasal bones and the mandibular fractures are the second most common fracture reported in the hospitalized pediatric trauma patients.^2^ Pediatric facial bones are more resistant to fracture due to their higher elasticity, poor pneumatization (by sinuses), thick surrounding adipose tissue and internally stabilized by the interrupted teeth in maxilla and mandible.

The incidence of fracture increases with age and peaks between 16 and 20 years. In pediatric patients the angle, condyle and the subcondylar region accounts for approximately 80% of the mandibular fractures. Symphysis and parasymphysis fracture accounts for 15 to 20%. Body fractures are rare.^3^

The purpose of this article is to provide an insight on mandibular fracture in pediatric patients and to assist the clinician in the management of this unique and highly specialized area of traumatology.

### Unique Features of the Pediatric Patient

Children have a higher surface-to-body volume ratio, metabolic rate, oxygen demand and cardiac output than adults. At birth, the ratio between cranial volume and facial volume is approximately 8:1. By the completion of growth, this ratio becomes 2.5:1.^4^ The retruded position of the face relative to the ‘protecting’ skull is an important reason for the lower incidence of midface and mandibular fractures and higher incidence of cranial injuries in young children (less than 5 years of age).^5,6^ With increasing age and facial growth, in a downward and forward direction, the midface and mandible become more prominent and the incidence of facial fractures increases, while cranial injuries decrease.^5^

Facial fractures in children occur less frequently than in adults and they are more often minimally displaced. This is because a thicker layer of adipose tissue covers the more elastic bones and the suture lines are flexible. In addition, stability is increased by the presence of tooth buds within the jaws and the lack of sinus pneumati-zation.^7-9^

In addition, children in the deciduous and mixed dentition stages demonstrate some capacity for spontaneous occlusal readjustment, after injury and treatment, as deciduous teeth are shed and permanent teeth erupt.

### Epidemiology of Facial Fractures in Children

Incidence

In children, the incidence and etiology of craniomaxillo-facial (CMF) trauma are also affected by age-related Activities, social, cultural and environmental factors. Overall, facial fractures in the pediatric population comprise less than 15% of all facial fractures.^10,11^ They are rare below age 5 (0.6-1.4%)^12,13^ and their incidence rises as children begin school.^7,10^

Another peak incidence occurs during puberty and adolescence with increased unsupervised physical activity and sports.^14,15^ Seasonal variations are reported with peak frequencies during summer months (except for skiing injuries) when outdoor activity is greatest.^7,16^

### Gender

The incidence of facial fractures is higher in boys than in girls worldwide and in all age groups. This male preponderance, which has remained constant over time, ranges from 1.1:1 to 8.5:1,^7,10,11,16^ and has been attributed to greater and more dangerous physical activities among boys.^7^ In younger age groups, gender differences are less significant and the etiologies are similar in both sexes.

### Etiology

Falls, sports-related injuries and road traffic accidents (RTA) constitute the most frequent causes of facial fractures in children.^7,14,16^ In infants and preschool children (up to age 6 years), falls in the home environment are the most common etiology of facial fractures.^16^ With increasing age and outdoor exposure, falls tend to occur outside the protected area of the home and parental supervision. As motor skills improve, sporting injuries become more common. Most sports related facial fractures occur in children 10 to 14 years of age. While young children usually sustain injuries from low velocity forces (e.g. falls), older children are more likely to be exposed to high-velocity forces (e.g. in RTA, sports-related trauma). High-velocity maxillofacial injuries in infants and young children may be under reported, because of their high mortality from concomitant neurocranial injuries.

The other main etiological factor is child abuse.^17^ Overall, the head, neck, face and mouth are involved in 50 to 75% of such cases. Victims of child abuse can be found in all age groups, but the groups most prone are newborns, infants and preschool children particularly boys. The perpetrators are parents or caretakers in 90% of the cases, especially in young children.^17^

### Site and Pattern

The site and pattern of a fracture depend on the interrelationship between etiology and force of the injury, and the unique anatomic features of the child’s stage of development. While infants (below age two) are more likely to sustain injuries of the frontal region, older children are more prone to injuries of the chin/lip region.

Children below age three usually sustain isolated, non-displaced fractures^7^ caused by low Impact/low-velocity forces. Common fractures in this age group are dentoalveolar, nasal and mandibular.

The condylar region is the most frequently fractured site^16^ being affected bilaterally in about 20% of pediatric patients.^9^ Fractures of the condyle are more common in children than in adults (50% of mandibular fractures *vs* 30%), because the highly vascularized pediatric condyle and thin neck are poorly resistant to impact forces during falls. In children below 6 years of age, condylar fractures are more often intracapsular than extracapsular in location.

Above this age, most condylar fractures occur in the neck region. Fractures in the condylar region are followed in number by symphysis, angle and body fractures, respectively.^16^ While body fractures are less common than in adults, symphysis and parasymphysis fractures of the mandible occur more often.

## MATERIALS AND METHODS

Six patients (aged between 2 and 5 years) who had para-symphysis fractures were selected for this study. Of these selected patients four were boys and two were girls. All the patients were conscious, not well oriented after the trauma. There was no any history of convulsion or vomiting. Radiographic orthopantogram (OPG) examination revealed parasymphysis fracture, which were complete, displaced and unfavorable in all these patients.

### Fabrication of MacLennan Splint (Figs 1 to 4)

The line of treatment was same for all the patients. MacLennan splint (acrylic cap splint) was planned because they were easy to fabricate and are frequently used in children as it is difficult to place circumferential mandibular stainless steel (SS) wires on the deciduous teeth due to their anatomic structure.

Two sets of upper and lower arch alginate impression were made and working models were prepared. After identifying the fracture line, the model was altered with the help of die cutting saw. The lower model was assembled against maxillary arch in occlusion and sealed with sticky wax. Base was made and models were articulated. MacLennan splint was made with cold cure acrylic [Dental Product of India (DPI)] with additional stabilization with co-axial SS wire. Then the splint was finished and polished and kept in antibacterial solution.

**Figs 1A to F:**
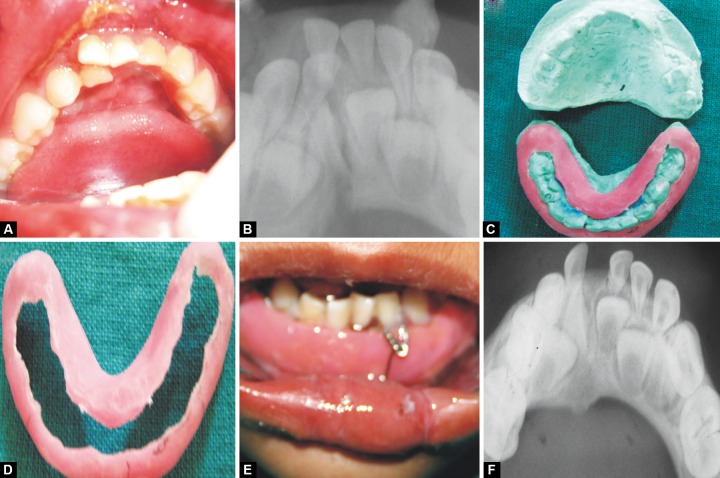
(A) Preoperative photograph, (B) preoperative radiograph, (C) fabrication of MacLennan splint, (D) fabricated MacLennan splint, (E) MacLennan splint with circummandibular wiring and (F) follow-up

**Figs 2A to F:**
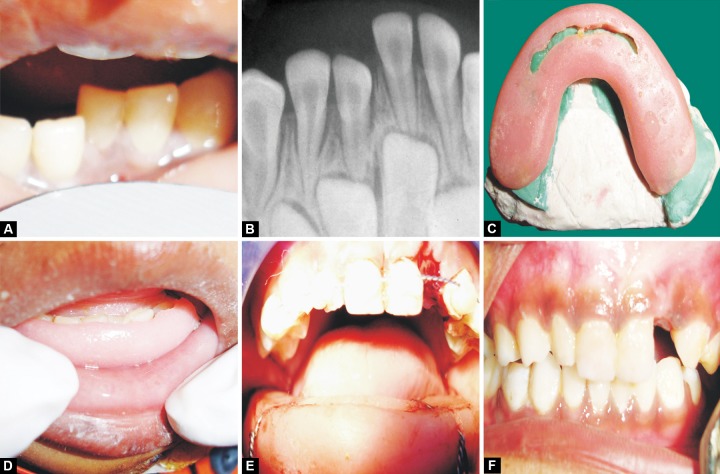
(A) Preoperative photograph, (B) preoperative radiograph, (C) fabrication of MacLennan splint, (D) MacLennan splint approximation, (E) MacLennan splint with circummandibular wiring and (F) follow-up

**Figs 3A to F:**
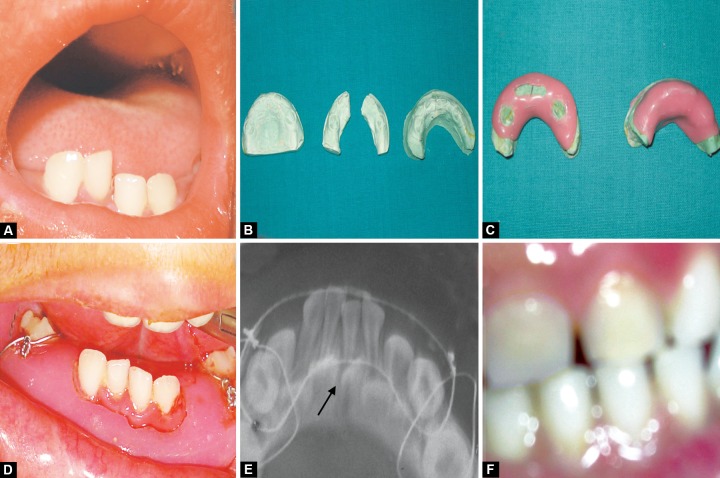
(A) Preoperative photograph, (B) fabrication of MacLennan splint, (C) fabricated MacLennan splint, (D) MacLennan splint with circummandibular wiring, (E) postoperative radiograph and (F) follow-up

**Figs 4A to D:**
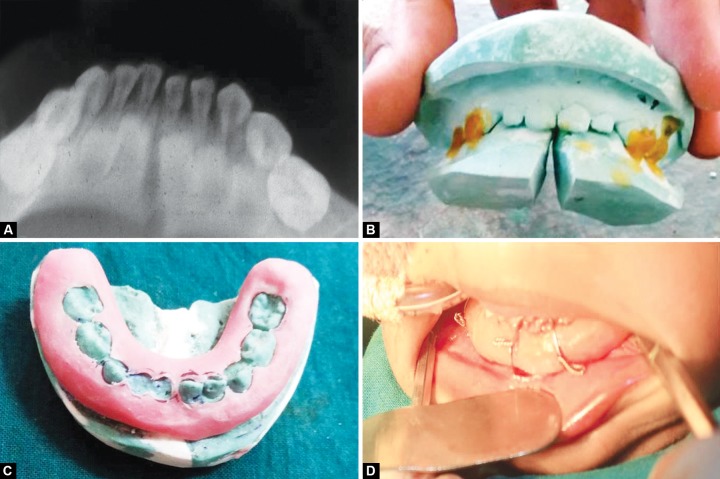
(A) Preoperative radiograph, (B) fabrication of MacLennan splint, (C) fabricated MacLennan splint, (D) MacLennan splint with circummandibular wiring

### Surgery under General Anesthesia

All the six patients were treated under general anesthesia. The mandibular arch was reduced and stabilized with prefabricated MacLennan splints and circum mandibular wiring (Figs 1 to 4) was done by placing small stab incision on the inferior border of the mandible on right-and left-side 4 to 5 mm from the midline. Mandibular awl was used to with first entry lingually along the body of the mandible by piercing lingual mucosa; after which the wire was passed onto buccal sulcus along the body of the mandible. Then the wire was held together and splint was stabilized by winding wire along the fractured segment. These splints were removed after 3 weeks in all the cases.

## RESULTS

There was very good outcome of these cases with MacLennan splint. The postoperative healing was good and uneventful. Postoperatively patients were observed for postoperative pain, swelling, malocclusion, soft lesions, eruption and healing. No complications were observed during postoperative recovery (Table 1). The radiographs were taken at different intervals which shows good amount of union between the fractured segments. Follow-up check-up was done for upto 1 year for all the cases. In two cases, we observed postoperative pain and swelling for a period of 1 week, which is insignificant. The patients were on medication postoperatively for a period of 5 days.

**Table 1:** **Table 1:** Patient data

*Patient*		*Sex*		*Age*		*Follow-up period*		*Postoperative pain*		*Postoperative swelling*		*Postoperative malocclusion*		*Postoperative eruption*		*Postoperative soft lesson*		*Postoperative healing*	
1.		F		2 years		1 year		N		N		N		Y		N		Good	
2.		M		4 years		1 year		Y		Y		N		Y		N		Good	
3.		M		4 years		1 year		N		N		N		Y		N		Good	
4.		M		3 years		1 year		N		Y		Y		Y		N		Good	
5.		M		3 years		1 year		Y		Y		N		Y		N		Good	
6		F		4 years		1 year		N		N		N		Y		N		Good	

N: No; Y: yes

## DISCUSSION

Pediatric maxillofacial fractures are not common and demonstrate different clinical features when compared with adults. They also need different treatment due to difference in their facial bones and skulls. Most of the pediatric fractures are firmly united in 2 to 3 weeks, because of the increased metabolic rate and increased osteogenic potential of periosteum in children.

Clinical signs and symptoms of pediatric fracture are the same as in adults. Thorough clinical examination, however, may be impossible in the uncooperative young trauma patient. Wide suture lines and the elasticity of the bone may mimic fracture gaps on palpation. Panoramic radiographs are the first step in all, but the very young patient but theses radiographs are less helpful, particularly in the mid-face region where poorly developed sinuses and tooth buds occupy space and obscure skeletal anatomic landmarks. Computed tomography, is the modality of choice. Computed tomography scans greatly increase diagnostic accuracy and have become the standard of care for imaging pediatric mid face trauma victims.

Treatment of mandibular fractures in children depends on the fracture site and the stage of skeletal and dental development. Tanaka N, Uchide N, Suzuki K (1993) stated that fractures of the mandible limited to the alveolar process are treated by open or closed reduction and immobilization by splints and arch bars for 2 to 3 weeks. Rarely, long-term mono-maxillary immobilization (via splinting) for upto 2 months is indicated to prevent malocclusion.

Mandibular fractures without displacement and malocclusion are managed by close observation, a liquid to soft diet, avoidance of physical activities (e.g. sports) and analgesics.

Displaced mandibular fractures need to be reduced and immobilized. When tooth buds within the mandible do not allow internal fixation with plates and screws^18^ this can be achieved with a mandibular splint fixed to the teeth by circum-mandibular wiring, gunning splint or a splint with MMF.

Displaced symphysis fractures can be treated by open reduction and rigid fixation through an intraoral incision after age six, when the permanent incisors have erupted. Open reduction internal fixation (ORIF) in parasymphysis fractures in feasible, when the buds of the canines have moved up from their inferior position at the mandibular border after age nine. Similarly, in body fractures, the inferior mandibular border can be plated, when the buds of the permanent premolar and molar have migrated superiorly toward the alveolus.^19^

Common recommended methods of management of mandibular fractures are as follows:

*0 to 2 years:* Treated as edentulous problems with Maclennan type of splint.

*2 to 4 years:* If well formed sound deciduous teeth eyelet wiring can be used. Cap splint.

*5 to 8 years:* MacLennan, Cap splints.

*9 to 11 years:* Cap Splints, arch bars, plating or trans-osseous wiring at lower border.

In this study, we preferred MacLennan type of cap splint because of various advantages like it covers both lingual and buccal cortical plates and hold the mandibular cortices securely. Other advantages include:

- Occlusion is open
- Function is not impaired
- Smaller adjustment or grinding can be done at the time of insertion
- Functional stresses increases remodeling
- Catabolic phase decrease.

### Prevention

The importance of preventive measures should be emphasized. Supervising adults, i.e. coaches, administrators, teachers and parents should be educated. Children should be encouraged to develop appropriate habits at an early age, because incidence and severity of sports-related injuries are inversely related to skill level and age.

Injuries in the children can be prevented by seat restraints, conventional seat belts, protective helmets, mouth guards, etc.

## CONCLUSION

While our follow-up period was too short and the patient population is too small to determine the long-term effects of fracture treatment with MacLennan splint, our preliminary impression is favorable. The splint showed sufficient rigidity and stability to enable initial bone healing of the mandible. Our observation showed that stability of the splint was high enough for fracture fixation. Tissue intolerance, growth restrictions, and occlusal abnormalities were not seen in our study and occlusal relationship could be restored in all cases. Benefits for children are evident since patient comfort is higher. However, potential problems relating to resorption and bone growth to be observed carefully and investigated in further clinical studies
